# Supplementary material for: “Where am I?” A snapshot of the developmental topographical disorientation among young Italian adults
Source: PLoS One. 2022 Jul 20;17(7):e0271334. doi: 10.1371/journal.pone.0271334 (PMC9299294; doi:10.1371/journal.pone.0271334)
Supplement: Attachment — Submitted filename: Rebuttal Letter Rev 2#.docx [file pone.0271334.s002.docx]

In the manuscript, all changes are highlighted in yellow.

PONE-D-22-02928R1

“Where am I?” A snapshot of the Developmental Topographical Disorientation among young Italian adults.

Journal Requirements:

Please review your reference list to ensure that it is complete and correct. If you have cited papers that have been retracted, please include the rationale for doing so in the manuscript text, or remove these references and replace them with relevant current references. Any changes to the reference list should be mentioned in the rebuttal letter that accompanies your revised manuscript. If you need to cite a retracted article, indicate the article’s retracted status in the References list and also include a citation and full reference for the retraction notice.

Reply: the reference list has been checked.

Additional Editor Comments:

Dear Dr. Piccardi,
you can see that the reviewers have now commented on your paper. One reviewer suggests accepting the paper as it stands now, while the other raises some concerns. By my own reading of the paper, I noticed that the manuscript has greatly improved. Therefore, I am encouraging you to revise the paper according to the instructions provided by the second reviewer.

Best wishes,

David Giofrè

Reviewers' comments:

Reviewer's Responses to Questions

**Comments to the Author**

1. If the authors have adequately addressed your comments raised in a previous round of review and you feel that this manuscript is now acceptable for publication, you may indicate that here to bypass the “Comments to the Author” section, enter your conflict of interest statement in the “Confidential to Editor” section, and submit your "Accept" recommendation.

Reviewer #1: All comments have been addressed

Reviewer #2: (No Response)

2. Is the manuscript technically sound, and do the data support the conclusions?

The manuscript must describe a technically sound piece of scientific research with data that supports the conclusions. Experiments must have been conducted rigorously, with appropriate controls, replication, and sample sizes. The conclusions must be drawn appropriately based on the data presented.

Reviewer #1: Yes

Reviewer #2: Partly

3. Has the statistical analysis been performed appropriately and rigorously?

Reviewer #1: Yes

Reviewer #2: Yes

4. Have the authors made all data underlying the findings in their manuscript fully available?

The [PLOS Data policy](http://www.plosone.org/static/policies.action#sharing) requires authors to make all data underlying the findings described in their manuscript fully available without restriction, with rare exception (please refer to the Data Availability Statement in the manuscript PDF file). The data should be provided as part of the manuscript or its supporting information, or deposited to a public repository. For example, in addition to summary statistics, the data points behind means, medians and variance measures should be available. If there are restrictions on publicly sharing data—e.g. participant privacy or use of data from a third party—those must be specified.

Reviewer #1: Yes

Reviewer #2: Yes

5. Is the manuscript presented in an intelligible fashion and written in standard English?

PLOS ONE does not copyedit accepted manuscripts, so the language in submitted articles must be clear, correct, and unambiguous. Any typographical or grammatical errors should be corrected at revision, so please note any specific errors here.

Reviewer #1: Yes

Reviewer #2: Yes

6. Review Comments to the Author

Please use the space provided to explain your answers to the questions above. You may also include additional comments for the author, including concerns about dual publication, research ethics, or publication ethics. (Please upload your review as an attachment if it exceeds 20,000 characters)

Reviewer #1: The authors replied all the questions raised. The manuscript can be accepted in the present form.

Reviewer #2: The authors have addressed almost all my previous concern sufficiently well.

I have a few additional comments.

- I found the description of the questionnaires a bit confusing. You present them as part of a unique “familiarity and spatial cognitive style scale” questionnaire (pages 8-9). If I am understanding, this questionnaire was used as a total score in other studies, while here you considered the SOD part as your dependent variable, and town knowledge, cognitive styles, left/right confusion, and means of transport as your predictors.

Reply: We would like to thank the Reviewer who allowed us to clarify the description of the instrument in the manuscript. First, we substituted the word “scale” for “questionnaire” and “subscales” instead of “sections”. We also add some more information about MoT and reliability of the subscales as asked by the Reviewer in the following concerns. Concerning the scale is always administered in its entirety and provides a total score, but it also provides scores for SOD; town knowledge; right/left confusion and spatial cognitive styles which were particularly useful in the present work to better describe the navigational skills of the population under investigation. For this reason, we considered these aspects in the analyses. They have also been taken into account in other works (e.g., Piccardi et al 2011; Nori and Piccardi 2012), while in other studies only SOD was considered in order to exclude the presence of individuals with navigational disorders from the sample.

Therefore, I suggest presenting the materials as follows
A) Anamnesis Questionnaire.
B) Demographic questionnaire (age, gender and educational level)
C) Means of transport questions (they are not numbered items of the scale, so I suppose they can be described as separately)
D) And then the “familiarity and spatial cognitive style scale” investigating in the order: sense of direction, left/right confusion, town knowledge, spatial cognitive styles.
- And for each part include all the information together (e.g., for the means of transport, you stated the items below in the text, but I would organize a dedicated paragraph in which finding all information about the measure, including scoring)

Reply: We have now divided the instruments into A (Anamnesis Questionnaire) and B (familiarity and spatial cognitive style scale). With respect to demographic information and means of transport are part of the scale and cannot be described separately. However, we have added information to help the reader better understand how they are articulated, then in the text we also refer to the scale that is in the appendix of the work of Piccardi et al 2011, which is the reason why we cannot include it in the supplementary materials of this work. We hope that the added information can make the scale clearer.

- For the Anamnesis Questionnaire could you add more explicitly what you asked the participants?

Reply: We add some more information about questions we asked participants

- Cronbach’s alphas of the subscales (SOD, town knowledge, spatial cognitive styles, ..) should be reported given the total score is not used here.

Reply: We now provided the internal consistencies of SOD and TK to compare the values with the original studies (i.e. Piccardi, Risetti and Nori 2011). Both in Nori and Piccardi (2012) and Piccardi, Risetti and Nori (2011) the only reliability measures concerned the total score and TK and SOD, but not the other subscales, for this reason, lacking previous comparative values, we did not proceed to report the internal consistency of spatial cognitive styles.

- Which is the rationale of considering the right/left dichotomous given data are collected in a 5-point Likert scale? Did the analyses differ when considering it not dichotomic?

Reply: We checked the Spearman correlation between the right/left confusion and SOD using RLC as a continuous variable, and we found a similar result (Rho= -.12; p< .001) obtained using dichotomic classification. This let us to assume that also the subsequent analyses reported in the paper yield similar results. However, we have decided to transform RLC from continuous to dichotomic because representing the presence/absence of a disorder the dichotomic subdivision makes stronger the result.

- Familiarity. In the first revision, I pointed out that “Furthermore, if this was the aim, the presentation of previous findings on the association between familiar environment knowledge and other factors with SOD merit to be better introduced.”
But I do not think the authors have addressed this point. Familiarity is only described as part of the Siegel and White model to reach survey knowledge, but neither specific rationale (supported by literature) for measuring it and hypothesis are presented.
Specifically, why consider town knowledge as a predictor of SOD? This could be ok, if supported.

Reply: We thank Reviewer for raising this point that we missed. We now explained better the reason why we insert TK as predictor, supporting our hypothesis with literature and a theoretical model.

But I was also wondering, why not add the town knowledge score together with SOD, given that DTD involves impaired orientation both in familiar and unfamiliar environments? (as you stated: “In general, people with DTD have normal memory and neuropsychological profiles, but show a major cognitive deficit in spatial cognition and complain of severe problems in navigation on an everyday basis. Specifically, they are unable to use cognitive maps or place-based navigation strategies to find their way around familiar and novel environments”).

Reply: We keep SOD and TK separate because they represent two independent albeit closely related constructs, i.e. it is true that increased familiarity with the environment produces positive effects on orientation, but as the Reviewer rightly points out in individuals with DTD the disorientation also occurs in highly familiar environments. It is now reasonable to see whether it can also represent a protective effect for them as we have now pointed out in the working hypothesis. Combining the two constructs, however, risks losing important information.
